# P-22. Implementing Patient-Reported Outcomes in Community Screening Programs: Results from the 2023 Southern Hemisphere Influenza Season

**DOI:** 10.1093/ofid/ofae631.230

**Published:** 2025-01-29

**Authors:** Jessie Edwards, Jocelyn Moyes, Erica Dueger, Nigel Stocks, Mvuyo Makhasi, Sheena Sullivan, Sibongile Walaza, Monique Chilver, Lana Allen, Daniel Blakeley, Fahima Moosa, Anne Von Gottberg, Nicole Wolter, Mignon Du Plessis, Cheryl Cohen, Richard Osborne, José Bartelt-Hofer

**Affiliations:** Discipline of General Practice, University of Adelaide, Australia, Adelaide, South Australia, Australia; Centre for Respiratory Diseases and Meningitis, National Institute for Communicable Diseases (NICD) of the National Health Laboratory Service, Johannesburg, South Africa, Johannesburg, Gauteng, South Africa; Sanofi Vaccines, Lyon France, Lyon, Rhone-Alpes, France; Discipline of General Practice, University of Adelaide, Australia, Adelaide, South Australia, Australia; Centre for Respiratory Diseases and Meningitis, National Institute for Communicable Diseases (NICD) of the National Health Laboratory Service, Johannesburg, South Africa, Johannesburg, Gauteng, South Africa; Department of Infectious Diseases, University of Melbourne, Melbourne, Victoria, Australia; Centre for Respiratory Diseases and Meningitis, National Institute for Communicable Diseases (NICD) of the National Health Laboratory Service, Johannesburg, South Africa, Johannesburg, Gauteng, South Africa; Discipline of General Practice, University of Adelaide, Australia, Adelaide, South Australia, Australia; Discipline of General Practice, University of Adelaide, Australia, Adelaide, South Australia, Australia; Discipline of General Practice, University of Adelaide, Australia, Adelaide, South Australia, Australia; Centre for Respiratory Diseases and Meningitis, National Institute for Communicable Diseases (NICD) of the National Health Laboratory Service, Johannesburg, South Africa, Johannesburg, Gauteng, South Africa; Centre for Respiratory Diseases and Meningitis, National Institute for Communicable Diseases (NICD) of the National Health Laboratory Service, Johannesburg, South Africa, Johannesburg, Gauteng, South Africa; Centre for Respiratory Diseases and Meningitis, National Institute for Communicable Diseases (NICD) of the National Health Laboratory Service, Johannesburg, South Africa, Johannesburg, Gauteng, South Africa; Centre for Respiratory Diseases and Meningitis, National Institute for Communicable Diseases (NICD) of the National Health Laboratory Service, Johannesburg, South Africa, Johannesburg, Gauteng, South Africa; Centre for Respiratory Disease and Meningitis, National Institute for Communicable Diseases, Johannesburg, South Africa., Johannesburg, Gauteng, South Africa; Measured Solutions for Health Pty. Ltd., Alphington, Australia, Alphington, Victoria, Australia; Sanofi Vaccines, Lyon France, Lyon, Rhone-Alpes, France

## Abstract

**Background:**

Vaccination against seasonal influenza can reduce the duration and severity of influenza symptoms and mitigate the negative impacts on quality of life (QoL). Patient reported outcomes (PROs) provide important data on the infected individual’s disease course. This study tested the Respiratory Infection, Intensity and Impact Questionnaire (RiiQ^TM^) PRO, in the context of community screening programs (CSP), to measure symptoms and QoL associated with influenza vaccination status, and provide lessons for future implementation.

**Table 1.**
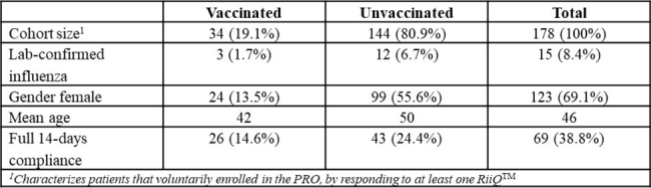
Respondent characteristics

**Methods:**

In 2023, two southern-hemisphere CSP in Australia (iSwab) and South Africa (Coughcheck) provided access to free respiratory illness diagnosis for patients with influenza-like-illness (ILI) via self-swabbing with laboratory confirmation (LCI), and invited participants to self-report the RiiQ^TM^ daily for 14 days, starting at enrollment. The RiiQ^TM^ measures symptoms (respiratory, systematic) and QoL (impact on: daily activities, emotions, others), with higher scores denoting worse symptoms/status. RiiQ^TM^ implementation differed between settings: iSwab responders completed digital- or paper-based forms and were remunerated; Coughcheck responders completed digital forms and were not remunerated. Pooled CSP, RiiQ^TM^ results comparing ILI vaccinated vs unvaccinated arms using the Area Under the Curve (AUC) were estimated.

**Table 2.**
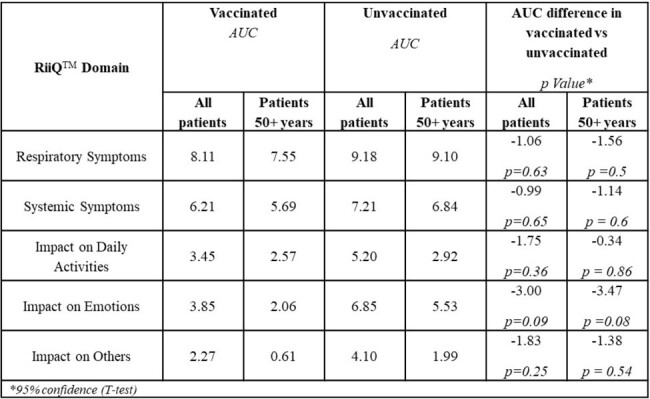
Comparison of RiiQ results over 14 days among vaccinated and unvaccinated respondents (Area under the curve (AUC) analysis)

**Results:**

178 CSP participants completed the RiiQ^TM^ (24% self-reported vaccinated, 8.4% total LCI). iSwab respondents preferred paper (70%) over digital surveys and saw higher RiiQ^TM^ compliance relative to Coughcheck (84.7% vs 16.7%). Vaccinated ILI individuals reported milder symptoms and better QoL across all 5 RiiQ^TM^ domains (lower AUC). Some differences were larger in those older than 50 years. Results were consistent in those with LCI but are not reported due to small sample size.

**Figure 1.**
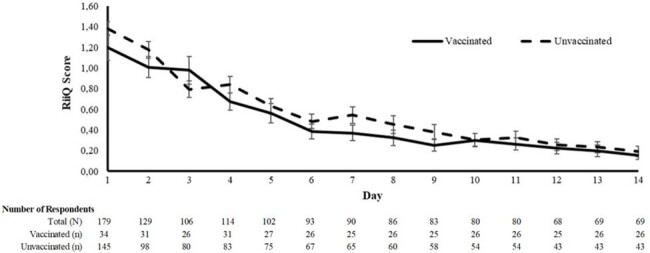
Systemic symptoms, vaccinated vs unvaccinated respondents

**Conclusion:**

CSP-based RiiQ^TM^, linked to LCI, is feasible and can provide useful information about the different symptoms and QoL of individuals when comparing vaccination status. Larger LCI samples are needed to confirm current RiiQ^TM^ results in ILI. Patient remuneration, active follow-up, and the availability of paper surveys improved participation and compliance.

**Figure 2.**
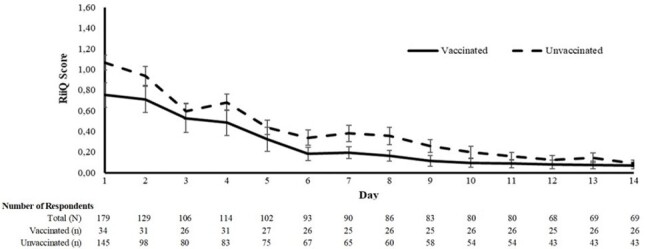
Impact on daily activities, vaccinated vs unvaccinated respondents

**Disclosures:**

**Jessie Edwards, Dr.**, Sanofi: Grant/Research Support **Jocelyn Moyes, Dr.**, Sanofi: Grant/Research Support **Erica Dueger, Dr.**, Sanofi: Employee|Sanofi: Stocks/Bonds (Private Company) **Nigel Stocks, Prof. Dr.**, Sanofi: Grant/Research Support **Mvuyo Makhasi, Dr.**, Sanofi: Grant/Research Support **Sheena Sullivan, Dr.**, Sanofi: Grant/Research Support **Sibongile Walaza, Dr.**, Sanofi: Grant/Research Support **Monique Chilver, MPH**, Sanofi: Grant/Research Support **Lana Allen, n/a**, Sanofi: Grant/Research Support **Daniel Blakeley, n/a**, Sanofi: Grant/Research Support **Fahima Moosa, n/a**, Sanofi: Grant/Research Support **Anne Von Gottberg, Prof. Dr.**, Sanofi: Grant/Research Support **Nicole Wolter, MD**, Sanofi: Grant/Research Support **Mignon Du Plessis, Dr.**, Sanofi: Grant/Research Support **Cheryl Cohen, n/a**, Sanofi: Grant/Research Support **Richard Osborne, Prof. Dr.**, Measured Solutions for Health Pty. Ltd.: Ownership Interest|Sanofi: Honoraria **José Bartelt-Hofer, Dr.**, Sanofi: Employee|Sanofi: Stocks/Bonds (Private Company)

